# Supplementary material for: Burdens of Apprentices Caused by the COVID-19 Pandemic and How They Deal with Them: A Qualitative Study Using Content Analysis One-Year Post-Breakout
Source: Healthcare (Basel). 2022 Nov 3;10(11):2206. doi: 10.3390/healthcare10112206 (PMC9691218; doi:10.3390/healthcare10112206)
Supplement: Supplementary file 1 [file healthcare-10-02206-s001.zip › healthcare-1974925-supplementary.pdf]

Table S1: Category System that Emerged from the Data of Question 1: “What burdens do you face as a result of the pandemic?”

Table S1. Category System that Emerged from the Date of Question 1: “What burdens do you face as a result of the pandemic?”

Categories	*n*	%
**Restrictions and Regulations**	**586**	**40.64**
Mask Requirement	190	13.18
Hobbies and Free Time	138	9.57
Curfew	130	9.02
Closed Retail and Gastronomy	99	6.87
Sports	70	4.85
Other COVID-19 Restrictions	55	3.81
Testing	31	2.15
Events	27	1.87
Constantly Changing Restrictions	13	0.90
Quarantine	6	0.42
**Social Life**	**532**	**36.89**
Limited Social Contact	477	33.08
Friends	280	19.42
Relatives of Family Members	62	4.30
Limited Social Contact in General	181	12.55
Family	48	3.33
Family in General	21	1.46
Arguments and Disagreements	18	1.25
Spatial Concentration	13	0.90
Social Losses	43	2.98
Break Ups/Impairments in Relationships	14	0.97
No Possibility to Make new Friends	13	0.90
Losing Friends	10	0.69
Concerns in the Team (Work-Related)	8	0.55
**Mental Burdens**	**474**	**32.87**
Lockdown-Related	188	13.04
Loneliness / Isolation	60	4.16
Boredom	40	2.77
Monotony	31	2.15
Feeling Deprived of Freedom	31	2.15
Feeling of Missing Out	27	1.87
Feeling Locked Up	17	1.18
Other Mental Burdens	**140**	**9.71**
Unspecified Other Mental Burdens	54	3.74
Fear of COVID-19 Infection	31	2.15

Fear of the Future	24	1.66
Fear of Punishment for Non-Compliance	18	1.25
Mental Burdens of Others	10	0.69
Deaths	10	0.69
News	8	0.55
Lack of Drive / Motivation	73	5.06
Depression	62	4.30
Stress	61	4.23
Mental Burden in General	60	4.16
Sleep	41	2.84
Anxiety	19	1.32
Self-Doubt	13	0.90
Concentration Difficulties	10	0.69
Sadness	8	0.55
Addiction	8	0.55
**Work and Education**	**328**	**22.75**
Distance Learning	90	6.24
Home Office	62	4.30
Working Conditions	55	3.81
Stress at School	39	2.70
Lack of Practice	35	2.43
Stress at Work	33	2.29
Conditions at School	30	2.08
Final Apprenticeship Examination / A-Levels	25	1.73
No Apprenticeship Position	23	1.60
Labor Market Uncertainties	9	0.62
Overload of Tasks	5	0.35
Furlough	3	0.21
Long Journey	3	0.21
**Physical Burdens**	**72**	**4.99**
Other Physical Complaints	35	2.43
Impaired Breathing	27	1.87
Headache	15	1.04
Postponement of Medical Treatments	4	0.28
Circulatory Complaints	3	0.21
No Burdens	81	5.62
Other Burdens	66	4.58
Don't Know	13	0.90
Not Like Before	11	0.76

Table S2: Category System that Emerged from the Data of Question 2: “What helps you to cope with these burdens?”

Table S2. Category System that Emerged from the Date of Question 2: “What helps you to cope with these burdens?”

Categories	*n*	%
**Social Contacts**	**526**	**36.48**
Friends	290	20.11
Staying in Touch / Communication	248	17.20
Family	152	10.54
Partner	85	5.89
Coworkers and Classmates	37	2.57
Teachers and Trainers	9	0.62
**Personal Attitudes**	**332**	**23.02**
Thinking / Staying Positive	57	3.95
Behavior Contrary to the Rules	38	2.64
Hope	38	2.64
Taking Breaks	34	2.36
Relaxing	30	2.08
Acceptance	29	2.01
Behavior in Compliance with Rules	26	1.80
Self	22	1.53
Suppression	20	1.39
Creating Structure	19	1.32
Studying	19	1.32
Problem Solving	18	1.25
Allowing Emotions	8	0.55
Motivation	6	0.42
Religion	5	0.35
**Leisure Activities**	**313**	**21.71**
Nature	129	8.95
Sports	123	8.53
Hobbies in General	53	3.68
Reading	19	1.32
Being Creative	16	1.11
Cooking and Eating	11	0.76
Learning Something New	10	0.69
Meditation	4	0.28
**Distraction**	**217**	**15.05**
Gaming	68	4.72
Music	68	4.72
TV	50	3.47
Distraction in General	44	3.05

Mobile Phone, Social Media, Internet	27	1.87
**Escapism**	**125**	**8.67**
Sleeping	39	2.70
Work	32	2.22
Alcohol	15	1.04
Drugs	15	1.04
Cigarettes	14	0.97
Escaping the Situation	10	0.69
Unhealthy Eating	8	0.55
Self-Harming	1	0.07
**Professional Help**	**38**	**2.64**
Psychotherapists	20	1.39
Medication	14	0.97
Psychologists	6	0.42
AMS / Other Labor Market Measures	2	0.14
Nothing	118	8.18
Other Resources	63	4.37
No Help Needed	42	2.91
Don't know	31	2.15
Pets	25	1.73
Wishful Thinking	15	1.04
